# Supplementary material for: Exploring determinants of hydrocele surgery coverage related to Lymphatic Filariasis in Nepal: An implementation research study
Source: PLoS One. 2021 Feb 26;16(2):e0244664. doi: 10.1371/journal.pone.0244664 (PMC7909642; doi:10.1371/journal.pone.0244664)
Supplement: S1 File — (DOCX) [file pone.0244664.s001.docx]

**IDI guideline with hydrocele patient (pre- surgery)**

District:

Municipality/rural municipality:

Date:

Code No:

**General Information of the respondent**

Name:

Ethnicity:

Age:

Religion:

Address:

Education:

Marital Status:

Relation with the head of HH………………………

Employment status……………………

Namaste (Short Introduction)

How are you today?

1. Let me know how and when did you get hydrocele? How long have you had hydrocele?
2. When did you know it was hydrocele?
3. How did you come to know?
4. Did you go for medical check-up? What did the health providers say about the diagnosis at the first time?
5. What do you think is the cause of hydrocele?
6. What is the causative agent?
7. How does it transmit to humans?
8. What is the treatment for hydrocele?
9. How long have you had hydrocele?
10. When did you know it was hydrocele?
11. How did you come to know?
12. Did you go for medical check-up? What did the health providers say about the diagnosis at the first time?
13. Have you ever been to the health facility for treatment and check-up regarding your condition?
14. If yes, can you tell me when and what happened while seeking treatment?
15. Within the last one-year did you go to the health providers for your hydrocele? What the health providers told you about your hydrocele/condition?
16. If not, what are the reasons (barriers) for not seeking treatment in health facility? (Do not read out the options. Probe when the respondents do not say anything)
17. Fear of being exposed to people, being looked down by people, staring, teasing
18. Lack of support from family members for seeking treatment,
19. Belief that the disease recovers on its own, result of sin from past life, hereditary
20. Faith in traditional healers,
21. Worried that the condition will worsen, impotency, fear of death
22. Distance to the health facility, no prompt service in health facility,
23. Difficulty in travelling due to hydrocele
24. Economic condition
25. Have you ever been approached or visited by any health worker regarding your condition?
26. If yes, who approached and when were you approached?
27. What did the health worker tell you?
28. Do you have information that hydrocele be treated with surgery and it is available free of cost in district hospital?
29. If yes, how did you come to know about this? Probe: friends, family, health worker, mass media
30. Can you explain what do you know about the available treatment?
31. If not, now that you know, would you be willing to go to the hospital to seek treatment?
32. If yes, when do you plan on going?
33. If no, why not? (probing: stigma, family support)
34. What kind of facilities (enablers) would be suitable for people like you so that you can access treatment in a timely manner? (please keep it open; do not mention the option)
35. What do information about treatment you need?
36. What about the distance of the service to your home?
37. How about the privacy in the health facility? What did you worry about the facility and its privacy?
38. What do you expect about the prompt service from the facilities/providers?
39. What kind of post-operative service do you need?
40. What kind of rehabilitation do you expect from the facilities?
41. Anything else that you would like to get so that you could get access to the service? Please mention ……..

Thank you so much for providing your valuable time for your interview. Would you be willing to answer if we call back again for more information? Thank you once again.

**IDI guideline with family member of hydrocele patient**

District:

Municipality/rural municipality

Date:

Code No.

**General Information of the respondent**

Name:

Ethnicity:

Age:

Religion:

Address:

Education:

Marital Status:

Relation with the head of HH………………… Relation with the patient………..

Namaste (Short Introduction)

1. What do you think is the cause of hydrocele?
2. What is the causative agent?
3. How does it transmit to humans?
4. What is the treatment for hydrocele?
5. How long did he (patient) have hydrocele?
6. When did you know it was hydrocele?
7. How did you come to know?
8. Have you ever taken him to the health facility for treatment and check-up regarding his condition?
9. If yes, can you tell me when and what happened while seeking treatment
10. What information did you get from the health providers regarding his condition, regarding the severity and seriousness, the impact for the patients, etc.?
11. Have you or the patient experienced barrier in seeking services from formal health facility? What are the barriers for not receiving service from the health facility?
12. Have you/ has patient feared of being exposed to people, being looked down by people, staring, teasing
13. Have you/has patient had lacked of support from family members for seeking treatment,
14. Have you/has patient believed that the disease recovers on its own, result of sin from past life, hereditary
15. Have you/has patient had faith in traditional healers?
16. Have you/has patient Worried that the condition will worsen, impotency, fear of death
17. Was the distance to the health facility preventing him/you for not seeking treatment?
18. Was “no prompt service in health facility” preventing him/you for not seeking treatment?
19. Was it the difficulty in travelling due to hydrocele that preventing him/you for not seeking treatment?
20. Was it the economic condition that preventing him/you for not seeking treatment?
21. Have you/has patient been informed that hydrocele can be treated with surgery? Have you/has patient been informed that the surgery is available free of cost in district hospital?
22. If yes, how did you come to know about this? Probe: friends, family, health worker, mass media
23. Can you explain what do you know about the available treatment?
24. Have you or anyone in the family ever been approached by any health worker regarding his condition?
25. If yes, when were you approached?
26. What information did you receive from health worker?
27. Would you be willing to take him to the hospital for seeking treatment if you know it can be treated?
28. If yes, when do you plan on going?
29. If no, why not?
30. What kind of facilities (enablers) do you think would be suitable for people like him, so that treatment can be accessed in a timely manner?
31. Information about treatment
32. Service availability closer to home
33. Privacy in HF
34. Prompt service
35. Rehabilitation

Thank you so much for providing your valuable time for your interview. Would you be willing to answer if we call back again for more information? Thank you once again.

**IDI guideline with hydrocele patient (post-surgery)**

District:

Municipality/rural municipality

Date:

Code No.

**General Information of the respondent**

Name:

Ethnicity:

Age:

Religion:

Address:

Education:

Marital Status:

Relation with the head of HH………………………

Employment status………………………

Namaste (Short Introduction)

I understand that you have had a hydrocele surgery, how are you today?

1. What do you think is the cause of hydrocele?
2. What is the causative agent?
3. How does it transmit to humans?
4. What is the treatment for hydrocele?
5. How long have you had hydrocele?
6. When did you know it was hydrocele?
7. How did you come to know?
8. When did you receive surgery for hydrocele?
9. Which hospital did you go for surgery?
10. Let me know how did you get the surgery? Was somebody/health provider approaching you to have the surgery or did you seek surgery to the hospital?
11. Can you explain about your experience regarding treatment facilities in the hospital?
12. Total cost, total days,
13. Experience during treatment and post-surgery
14. Did you have information that hydrocele can be treated with surgery and it is available free of cost in district hospital?
15. How did you come to know about surgery?
16. Source of information, health worker, friends, family
17. What do you think are the reasons (barriers) for those who do not seeking treatment in health facility? (do not read/mention the options) 🡪 it should be genuinely told by the informant. You may mention them if the informant did not say anything to answer
18. Fear of being exposed to people, being looked down by people, staring, teasing
19. Lack of support from family members for seeking treatment,
20. Belief that the disease recovers on its own, result of sin from past life, hereditary
21. Faith in traditional healers,
22. Worried that the condition will worsen, impotency, fear of death
23. Distance to the health facility, no prompt service in health facility,
24. Difficulty in travelling due to hydrocele
25. Economic condition
26. How do you think your life has improved post-surgery?
27. Mobility
28. Employment and income
29. Confidence

Thank you so much for providing your valuable time for your interview. Would you be willing to answer if we call back again for more information? Thank you once again.

**KII guideline with focal person at the central level**

District:

Municipality/rural municipality

Date:

Code No.

**General Information of the respondent:**

Name:

Designation:

Institution:

Experience of in the current organization:

Experience in the current position:

Namaste (Short Introduction)

1. Could you tell me about the Lymphatic Filariasis program in Nepal, especially the MMDP component?
2. Could you tell me about hydrocele surgery facility? Probe: Hospitals designated for surgery, doctors orientation/training
3. What facilities are included in the program? (probe: transportation, accommodation during hospitalization, care taker allowance, additional facilities post-surgery)
4. How long has the program (hydrocele surgery) being run at the national level?
5. How many people benefitted so far from the service?
6. Is there any target set by the government to achieve beneficiaries (of hydrocele)?
7. Elimination 2020
8. Post elimination strategy
9. What is the mechanism for a patient being referred to the hospital for getting service? How did you approach the patient and the family so they are willing to get surgery?
10. As a program implementer, what could be the barriers for patients utilizing the service provided free of cost by the government? And enablers? (probe for: information dissemination, lack of awareness, social stigma, poverty, distance)
    1. Barriers from patients and families or communities?
    2. Barriers from the program implementers?
    3. Barriers from the health system and management?
    4. Barriers from the policy or central levels?
11. Is there any instances where service is compromised?
12. lack of funding, lack of guideline, lack of coordination?
13. Is approaching patients and families difficult for the program? Could you please explain it to me?
14. What are the activities done from central level to ensure that the target population get the right information on time and receives the services available?
15. Collaboration with the media for information dissemination
16. Coordination with the private hospitals for providing surgery
17. Doctors/surgeon training/refresher training
18. Community health workers training, orientation for case identification
19. Finally, do you have any suggestion for improving the hydrocele surgery rates so that all the target population benefit from the service?

**KII guideline with focal person at district health office**

District:

Municipality/rural municipality

Date:

Code No.

**General Information of the respondent:**

Name:

Designation:

Institution:

Experience of in the current organization:

Experience in the current position:

Namaste (Short Introduction)

1. Could you tell me about the Lymphatic Filariasis program in the district, especially the MMDP component?
2. Could you tell me about hydrocele surgery facility?
3. Hospitals designated for surgery,
4. Doctors/HW orientation/training
5. Budget allocation
6. What facilities are included in the program? (probe: transportation cost, accommodation during hospitalization, care takers allowance, additional facility post-surgery)
7. How long has the program (hydrocele surgery) being run in the district?
8. Do you have records of how many people benefitted so far from the service?
9. Is there any target set by the district to achieve beneficiaries?
10. Is there any mechanism of patient being referred to the district hospital for getting service?
11. As a program implementer in a district, what could be the barriers for patients utilizing the service provided free of cost by the government? And enablers?
12. Lack of IEC materials for proper information dissemination
13. High case load of patients with other illnesses
14. Geographical constraints for patients
15. Challenges in identifying eligible cases for surgery etc.
16. Are there any instances where service is compromised?
17. Lack of funding,
18. Lack of guideline,
19. Lack of coordination
20. What additional activities are done at the district to ensure that the target population gets the right information on time and receives the services available?
21. Finally, do you have any suggestion for improving the hydrocele surgery rates or reaching the target population so that all benefit from the service?

**KII guideline with the district hospital focal person**

District:

Municipality/rural municipality:

Name of the hospital

Date:

Code No.

**General Information of the respondent:**

Name:

Designation:

Institution:

Experience of in the current organization:

Experience in the current position:

Namaste (Short Introduction)

1. Could you tell me about the services for hydrocele patient in the hospital?
2. How long has it been since the hospital started providing hydrocele surgery facilities?
3. What facilities are included in the program? (probe: transportation, accommodation during hospitalization, post-surgery facilities, rehabilitation)
4. Do you have records of how many people benefitted so far from the service from the hospital?
5. What is the mechanism for a patient being referred to the hospital for getting service?

a. Referral slip from local HF

b. Proof of diagnosis of hydrocele due to LF

1. As a program implementer, what could be the barriers for patients utilizing the service provided free of cost? And enablers?
2. Unavailability of surgical instruments,
3. Lack of IEC materials for counselling,
4. High case load of patients with other illnesses,
5. Lack of trainer doctors, lack of training from the government
6. Post-operative management challenges,
7. Challenges in identifying eligible cases for surgery
8. Geographic constraints for patients
9. Are there any instances where service is compromised?
10. Lack of funding, guideline, coordination
11. Lack of trained doctors, vacant post, lack of incentives for doctors
12. What are the activities done from hospital to ensure that the target population get the right information on time and receives the services available?
13. Collaboration with the media for information dissemination
14. Coordination with private hospitals,
15. Local health workers training, orientation for active case finding and referral
16. Finally, do you have any suggestion for improving the hydrocele surgery rates so that all the target population benefit from the service?

**FGD guideline with female community health volunteers**

District:

Municipality/rural municipality

Ward No:

Date:

Code No.:

Facilitators welcome and introduction and instructions to the participants.

Welcome and thank you for agreeing to participate in this discussion. You have been asked to participate as your point of view is important. The discussion will take nearly one hour. If you agree, I would like to tape the discussion for better capturing your views. However, please note that the information you provided will be confidential and the discussion will be anonymous.

**Ground Rules:**

- Please try to comment accurately and truthfully as possible.
- Speak once at a time. Participate equally. There is no particular order for speaking but let other finish speaking before expressing your views.
- There is no right or wrong answer. Each person is entitled to their own views and opinions, you do not necessarily have to agree and disagree with anyone.

1. Can you provide brief information about hydrocele cases in your areas? (number of cases, untreated cases)
2. What do you know about the treatment facility by the government regarding hydrocele?
3. Have you received any training or orientation from the heath facility regarding identifying, counselling and referring patient to health facility? Probe: When, how was the training?
4. Have you ever counselled or referred a patient to hospital for receiving treatment? Probe: How do you convince the patient, how often, success rate
5. Generally, what is the socio-cultural view towards the person with hydrocele and the condition itself?
6. Societal and family treatment of the patient, stigmas such as shame to participate socially, impotency, disease transmission to another person, neglect from family members, marriage problems between spouses, marriage prospects of a patient
7. Health care treatment seeking practice such as belief in faith healers, shamans
8. Belief such as curse from god for the sins of past life, hereditary disease
9. What do you think are major barriers for a patient to seek treatment from health facilities? What are the enablers?
10. Lack of family support, patients do not want to speak openly about illness, gender issues in diagnosis, feeling of embarrassment by the patient or the family members
11. patient viewed as a burden to the family
12. Geographic constraints for accessing the service
13. Economic condition
14. Stigma such as impotency after surgery,
15. Belief that the disease cannot be treated, fear of death from surgery
16. In your opinion, what should be done in order to provide more effective service to the person with hydrocele?
17. Community awareness activities
18. Incentives like transportation facilities
19. Rehabilitation services
20. How do you think you can help the hydrocele patient to refer to the health facility for getting necessary treatment? What is your role?
21. Counselling
22. Incentive for you for referring patient
23. Training for counseling and referring
