# Supplementary material for: Exploring determinants of hydrocele surgery coverage related to Lymphatic Filariasis in Nepal: An implementation research study
Source: PLoS One. 2021 Feb 26;16(2):e0244664. doi: 10.1371/journal.pone.0244664 (PMC7909642; doi:10.1371/journal.pone.0244664)
Supplement: S2 File — (DOCX) [file pone.0244664.s002.docx]

**xfO{8«f];Ln ePsf JoQmLx?nfO{ -zNos[of k'j{_ ;f]lwg] IDI lgb{]lzsf**

lhNnf M

gu/kflnsf÷ufpFkflnsf M

ldlt M

sf]8 g+= M

pQ/bftfsf] ;fwf/0f hfgsf/L M

gfd M

hfltotf M

pd]/ M

wd{ M

7]ufgf M

lzIff M

j}jflxs cj:yf M

3/d'nL;Fusf] gftf M

/f]huf/sf] cj:yf M

**kl/ro**

!_ tkfO{nfO{ cfh s:tf] 5 <

- tkfO{nfO{ xfO{8«f];Ln ePsf] slt eof]<
- tkfO{nfO{ of] xfO{8«f];Ln xf] eg]/ slxn] yfxf eof] <
- tkfO{n] s;/L yfxf kfpg' eof] <

@_ tkfO{ slxNo} cfˆgf] ;d:of lnP/ :jf:Yo ;+:yfdf pkrf/sf] nflu hfg'ePsf] 5 <

- obL 5 eg], slxn] hfg'eof] / pkrf/ ug{ hfg' x'Fbf :jf:Yo ;]jf k|bfosn] tkfO{FnfO{ xfO{8«f];Lnsf] af/]df s] eGg' ePsf] lyof] <
- obL 5}g eg] lsg hfg'ePg <

#_ tkfO{sf] ljrf/df xfO{8«f];Lnsf sf/0fx? s]-s] x'g\ <

- of] dflg;df s;/L ;5{ -transmit_ <
- xfO{8«f];Lnsf] pkrf/ s] xf]<

$_ tkfO{sf] ljrf/df xfO{8«f];Ln ePsf JoQmLx?n] :jf:Yo ;+:yfdf pkrf/ ug{ ghfg'sf sf/0f / jfwfx? s]-s] x'g\ < (pQ/x? k9]/ g;'gfpg'xf];\ . pQ/bftfn] s]xL geg]sf] cj:yfdf dfq k|f]a ug{'xf];\)

- cGo JoQmLx?n] yfxf kfpg] 8/n], cGo JoQmLx?n] tNnf] gh/n] x]g]{ eP/, 3f]l/P/ x]/L/xg] jf lh:Sofpg] eP/
- pkrf/ lngsf] nflu 3/kl/jf/sf ;b:ox?af6 kof{Kt ;xof]u gePsfn]
- /f]u cfFkm} lgoGq0fdf cfpg] ;fF]r]/, k'j{ hGdsf] kfksf] k/L0ffd, j+zf0f'ut\
- wfdL emfFqmLdf ljZjf;
- cj:yf emg} lau|g] ;f]rfO{ cfPsf], zNos[of ub{f k'?iff]Tj u'Dg] jf d[To'sf] eon]
- :jf:Yo ;+:yf 6f9f kg]{ ePsf]n], :jf:Yo ;+:yfdf tTsfn :jf:Yo ;]jf gkfpg] ePsf]n]
- xfO{8«f];Lnn] ubf{ lxF88'n ug{ s7Lg eP/
- cfly{s ;d:of

%_ s] tkfO{sf] :jf:Yo ;d:ofnfO{ lnP/ s'g} klg :jf:Yo sd{rf/Ln] tkfO{nfO{ ;Dks{ ug{'ePsf] jf e]6\gcfpg' ePsf] 5 <

- obL 5 eg], tkfO{nfO{ s;n] / slxn] ;Dks{ ug{'eof] <
- :jf:Yo sd{rf/Ln] tkfO{nfO{ s] eGg'eof] <

^_ xfO{8«f];Lnsf] pkrf/ zNos[ofaf6 x'g] / of] lhNnf c:ktfnaf6 lgMz'Ns k|fKt ug{ ;lsg] af/]df tkfO{nfO{ s]xL hfgsf/L 5 <

- obL 5 eg], tkfO{n] of] hfgsf/L s;/L yfxf kfpg' eof] < k|f]a M ;fyL , kl/jf/ , :jf:Yo sd{rf/L, ;~rf/ dfWod jf c? s'g} dfWod
- tkfO{nfO{ pknAw ;]jfx? af/] s]-s] yfxf 5 , atfOlbg'x'G5 <

&_ o;sf] pkrf/ ug{ ldNg] yfxf ePdf tkfO{ pkrf/ ug{ :jf:Yo ;+:yf hfg'x'G5 <

- obL hfg'x'G5 eg], tkfO{ slxn] hfg] ;f]Frdf x'g'x'G5 <
- obL hfg'x'Gg eg], lsg hfg'x'Gg < k|f]a M k|Zg g+ $ df pNn]v u/]sf] k|f]ax? k'g k|of]u ug{'xf];\

*_ tkfO{h:tf] xfO{8«f];Lnaf6 lkl8t JoQmLx?nfO{ ;dod} pkrf/sf] kx'+r k'of{pgsf] nfuL s]-s:tf ;]jf;'ljwfx? pko'Qm x'G5g\ < (s[kof pQ/x? k9]/ g;'gfpg'xf];\)

- tkfO{nfO{ rflxPsf] pkrf/sf] af/]df s]-s:tf hfgsf/Lx? kfpg'eof] eg] tkfO{nfO{ ;lhnf] x'GYof] <
- jf;:yfg glhs} ;]jfsf] pknAwtf
- :jf:Yo ;]jfdf uf]kgLotf , tTsfn ;]jf
- zNos[of kZrftsf ;]jfx?
- k'gM:yfkgf

(_ :jf:Yo ;]jfsf] kx'+rdf k'Ug jf cGo s]xL rflxPsf] jf eGg rfxg'x'G5 < s[kof atfO{lbg'xf];\

cGt/jftf{sf] nflu cfˆgf] ;do lbg'ePsf]df tkfO{nfO{ w]/}-w]/} wGojfb lbg rfxG5' . d}n] s]xL yk hfgsf/L kfpg k'gM ;Dks{ u/]df s'/fsfgL ug{ O{R5's x'g'x'G5 < tkfO{nfO{ k'gM wGojfb .

**xfO{8«f];Ln ePsf JoQmLsf kl/jf/;Fu ;f]lwg] IDI lgb{]lzsf**

lhNnf M

gu/kflnsf ÷ ufpFkflnsf M

ldlt M

sf]8 g+= M

pQ/bftfsf] ;fwf/0f hfgsf/L M

gfd M

hfltotf M

pd]/ M

wd{ M

7]ufgf M

lzIff M

j}jflxs cj:yf M

3/d'nL;Fusf] gftf M

xfO{8«f];Ln ePsf JoQmL;Fusf] gftf M

**kl/ro**

!_ tkfO{sf] ljrf/df xfO{8«f];Lnsf sf/0fx? s]-s] x'g\ <

- of] dflg;df s;/L ;5{ -transmit_ <
- xfO{8«f];Lnsf] pkrf/ s] xf]<

@_ tkfO{sf] ……………………………….. nfO{ xfO{8«f];Ln ePsf] slt eof]<

- tkfO{nfO{ Tof] xfO{8«f];Ln xf] eg]/ slxn] yfxf eof] <
- tkfO{n] s;/L yfxf kfpg' eof] <

#_ tkfO{ slxNo} ………………………….. nfO{ jxfFsf] ;d:ofnfO{ lnP/ :jf:Yo ;+:yfdf pkrf/ / lrlsT;f hfFrsf] nflu hfg'ePsf] 5 <

- obL 5 eg], s] tkfO{ dnfO{ pkrf/ kfpg slxn] hfg'eof] / pkrf/ kfpg] qmddf s] eof] eGg ;Sg'x'G5 <
- tkfO{n] :jf:Yo ;]jf k|bfosaf6 jxfFsf] cj:yf, /f]u slQsf] uDeL/ / lrGtfhgs /x]sf], la/fdLnfO{ k/]sf] c;/ cflb af/] s:tf] k|sf/sf] hfgsf/Lx? k|fKt ug{'eof] <

$_ tkfO{sf] ljrf/df, tkfO{ jf tkfO{sf] ………………………….. n], :jf:Yo ;+:yfaf6 ;]jf k|fKt ug{ s]xL afwfx?sf] ;fdgf ug{'ePsf] lyof] < cyjf xfO{8«f];Ln ePsf JoQmLx?n] :jf:Yo ;+:yfdf pkrf/ ug{ ghfg'sf sf/0f / jfwfx? s]-s] 5g <

- cGo JoQmLx?n] yfxf kfpg] 8/n], cGo JoQmLx?n] tNnf] gh/n] x]g]{ eP/ , 3f]l/P/ x]/L/xg] jf lh:Sofpg] eP/
- pkrf/ lngsf] nflu 3/kl/jf/sf ;b:ox?af6 kof{Kt ;xof]u gePsfn]
- /f]u cfFkm} lgoGq0fdf cfpg] ;f]Fr]/, k'j{ hGdsf] kfksf] kl/0ffd , j+zf0f'ut\
- wfdL emfFqmLdf ljZjf;
- cj:yf emg} lau|g] ;f]rfO{ cfPsf], k'?iff]Tj u'Dg] jf d[To'sf] eon]
- :jf:Yo ;+:yf 6f9f kg]{ ePsf]n], tTsfn :jf:Yo ;]jf gkfpg] ePsf]n]
- xfO{8«f];Lnn] ubf{ lx88'n ug{ s7Lg eP/
- cfly{s ;d:of

%_ xfO{8«f];Lnsf] pkrf/ zNos[ofaf6 x'g] / of] lhNnf c:ktfn af6 lgz'Ns k|fKt ug{ ;lsg] af/]df tkfO{nfO{ s]xL hfgsf/L 5 <

- obL 5 eg], tkfO{n] of] hfgsf/L s;/L yfxf kfpg' eof] < k|f]a M ;fyL , kl/jf/ , :jf:Yo sd{rf/L, ;+rf/ dfWod
- tkfO{nfO{ pknAw ;]jfx? af/] s]-s] yfxf 5 , atfOlbg'x'G5 <

^_ s] jxfFsf] :jf:Yo ;d:ofnfO{ lnP/ s'g} klg :jf:Yo sd{rf/Ln] tkfO{ jf tkfO{sf] 3/sf] cGo s'g} klg ;b:onfO{ ;Dks{ ug{'ePsf] 5 <

- obL 5 eg], tkfO{nfO{ s;n] / slxn] ;Dks{ ug{'eof] <
- :jf:Yo sd{rf/Ln] tkfO{nfO{ s]-s:tf hfgsf/Lx? lbg'eof] <

&_ o;sf] pkrf/ ug{ ldNg] yfxf ePdf tkfO{ jxfFnfO{ pkrf/ u/fpg :jf:Yo ;+:yf hfg O{R5's x'g'x'G5 <

- obL hfg'x'G5 eg], tkfO{ slxn] hfg] ;fF]rdf x'g'x'G5 <
- obL ghfg] eP, lsg hfg'x'Gg <

*_ tkfO{sf] ljrf/df jxfFh:tf] JoQmLnfO{ ;dod} pkrf/sf] kx'+r k'of{pgsf] nflu s]-s:tf ;]jf;'ljwfx? pko'Qm x'G5g\ <

- pkrf/sf] af/]df hfgsf/L
- jf;:yfg glhs} ;]jfsf] pknAwtf
- :jf:Yo ;]jfdf uf]kgLotf , tTsfn ;]jf
- zNos[of kZrftsf ;]jfx?
- k'g:yf{kgf

cGtjf{tf{sf] nflu cfˆgf] ;do lbg'ePsf]df tkfO{nfO{ w]/}-w]/} wGojfb lbg rfxG5' . d}n] s]xL yk hfgsf/L kfpg k'gM ;Dks{ u/]df s'/fsfgL ug{ O{R5's x'g'x'G5 < tkfO{+nfO{ k'gM wGojfb .

**xfO{8«f];Ln** **ePsf JoQmLx?nfO{ -zNos[of kZrft_ ;f]lwg] IDI lgb{]lzsf**

lhNnf M

gu/kflnsf ÷ ufpFkflnsf M

ldlt M

sf]8 g+= M

pQ/bftfsf] ;fwf/0f hfgsf/L M

gfd M

hfltotf M

pd]/ M

wd{ M

7]ufgf M

lzIff M

j}jflxs cj:yf M

3/d'nL;Fusf] gftf M

/f]huf/sf] cj:yf M

**kl/ro**

!_ tkfO{sf] ljrf/df xfO{8«f];Lnsf sf/0fx? s]-s] x'g <

- o;sf sf/sx? s]-s] x'g\ <
- of] dflg;df s;/L km}lnG5 <
- xfO{8«f];Lnsf] pkrf/ s] xf] <

@_ tkfO{nfO{ xfO{8«f];Ln ePsf] stL eof] <

- tkfO{nfO{ Tof] xfO{8«f];Ln xf] eg]/ slxn] yfxf eof] <
- tkfO{n] xfO{8«f];Ln ePsf] s;/L yfxf kfpg' eof] <

#_ tkfO{n] xfO{8«f];Lnsf] zNos[of slxn] u/fpg'eof] < tkfOn] zNos[of s;/L kfpg'eof] atfO{ lbg'x'G5 <

- tkfO{ zNos[ofsf] nflu s'g c:ktfn hfg'eof] <
- s] tkfO{nfO{ :jf:Yo k|bfos cfkm}n] jf c? s;}n] zNos[of ug{sf] nflu ;Dks{ ug{'eof] jf tkfO{ cfkm} c:ktfn uP/ ;]jf vf]Hg'eof] <

$_ c:kftndf ;]jf ;'ljwfx? kfpFbfsf] cg'ej dnfO{ atfOlbg'xf];\ g <

- s'n vr{, hDdf lbg
- pkrf/sf] a]nfdf / zNos[of kl5sf cg'ejx?

%_ xfO{8«f];Lnsf] pkrf/ zNos[ofaf6 x'g] / of] lhNnf c:ktfn af6 lgMz'Ns k|fKt ug{ ;lsg] af/]df tkfO{+nfO{ s]xL hfgsf/L 5 <

^_ zNos[ofsf] af/]df tkfO{n] s;/L yfxf kfpg'eof] <

- :jf:Yo sd{rf/L, kl/jf/sf ;b:o , ;fyLx? af6 hfgsf/L

&_ xfO{8«f];Ln ePsf JoQmLx?n] :jf:Yo ;+:yfdf pkrf/ ug{ ghfg'sf sf/0f / jfwfx? s]-s] x'g\ < (s[kof pQ/x? k9]/ g;'gfpg'xf];\)

- cGo JoQmLx?n] yfxf kfpg] 8/n], cGo JoQmLx?n] tNnf] gh/n] x]g]{ eP/, 3f]l/P/ x]/L/xg] jf lh:Sofpg] eP/
- pkrf/ lngsf] nflu 3/kl/jf/sf ;b:ox?af6 kof{Kt ;xof]u gePsfn]
- /f]u cfFkm} lgoGq0fdf cfpg] ;f]r]/, k'j{ hGdsf] kfksf] kf/L0ffd, jGzfg'ut
- wfdL emfFqmLdf ljZjf;
- cj:yf emg} lau|g] ;f]rfO cfPsf], k'?iff]Tj u'Dg] jf d[To'sf] eon]
- :jf:Yo ;+:yf 6f9f kg]{ ePsf]n], tTsfn :jf:Yo ;]jf gkfpg] ePsf]n]
- xfO{8«f];Lnn] ubf{ lxF88'n ug{ s7Lg eP/
- cfly{s ;d:of

*_ zNos[of kZrft tkfO{nfO{ s;/L cfˆgf] b}lgsLdf ;'wf/ ePsf] h:tf] nfU5 <

- lxF88'n ug{
- /f]huf/ / cfly{s cj:yf
- cfTdljZjf;

cGt/jftf{sf] nflu cfˆgf] ;do lbg'ePsf]df tkfO{nfO{ w]/}-w]/} wGojfb lbg rfxG5' . d}n] s]xL c? hfgsf/L kfpg k'gM ;Dks{ u/]df s'/fsfgL ug{ O{R5's x'g'x'G5 < tkfO{nfO{ k'gM wGojfb .

**Olk8]ldof]nf]hL tyf /f]u lgoGq0f ljefusf s]lG›o JoQmL;Fusf] KII lgb{]lzsf**

lhNnf M

gu/kflnsf÷ufpFkflnsf M

ldlt M

sf]8 g+= M

pQ/bftfsf] ;fwf/0f hfgsf/L M

kb M

;+:yfsf] gfd M

xfnsf] ;+:yfdf cg'ej M

xfnsf] kbdf cg'ej M

**kl/ro**

!_ s] tkfO{ dnfO{ g]kfndf lnDˆol6s lkmnfl/ol;;, d'Voto MMDP sfo{qmdsf] af/]df atfO{ lbg'x'G5 <

@_ s] tkfO{ dnfO{ xfO{8«f];Ln zNos[of ;'ljwfsf] af/]df atfO{ lbg'x'G5 < k|f]a M zNos[ofsf nflu tf]lsPsf c:ktfnx?, 8fS6/ tyf :jf:y sfo{st{fx?nfO{ cfjZos tflnd

#_ o;\ sfo{qmddf s]-s:tf ;'ljwfx? ;dfj]z 5g\ <

k|f]a M oftfoft ;'ljwf, pkrf/sf] qmddf cfjf;sf] Joj:yf, x]/rfx ug]{nfO{ eQf, tTsfn zNos[of kZrft\ cGo ;'ljwfx?

$_ /fi6«Lo:t/df xfO{8«f];Ln zNos[of sfo{qmd ;~rfngdf cfPsf] slt eof] <

%_ xfn;Dd slthgfn] o; ;'ljwf k|fKt ul/;s]sf 5g\ / s'n cg'dflgt\ nfe ;+Vof slt xf]<

^_ ;/sf/n] s'n cg'dflgt\ nfe lng] JoQmLx?sf] ;+Vof k|fKt ug{ s'g} /0fgLltx? agfPsf] 5 <

- pGd'ng @)@) nIo
- pGd'ng kl5sf /0fgLltx?

&_ xfO{8«f];Ln zNos[of ;]jfsf] nflu la/fdLnfO{ c:ktfndf k|]if0f ul/g] sfo{ljwL s:tf] /x]sf] 5 < h:t} la/fdL jf la/fdLsf] kl/jf/nfO{ s;/L zNolqmof ug{sf] nflu ;Dks{ ul/G5 <

*_ sfo{Gjogstf{ ePsf] gftfn] x]g{'kbf{, la/fdLnfO{ ;/sf/n] lgMz'Ns k|bfg u/]sf] ;]jfsf] kx'+rdf k'Ug s]-s:tf afwfx? cfO{k/]sf 5g\ < / k|f]T;fxg\sf nflu s]-s:tf sfo{qmdx? ;~rfng\ ul/Psf 5g\ <

- ;'rgfsf] k|rf/ k|;f/, hfu?stfsf] sdL, ;fdflhs nf~5gf, ul/jL, b'/L
- la/fdL, kl/jf/ / ;d'bfoaf6
- sfo{qmd sfof{Gjfogstf{x? af6
- :jf:Yo k|0ffnL / Joj:yfkg
- gLlt tyf s]G›Lo txaf6

(_ xfO{8«f];Ln zNos[of sfo{qmd ;~rfng ePkZrft\ ;]jfdf slxNo} ;Demf}tf ug{'k/]sf] s'g} cj:yf cfOk/]sf] 5 <

- cfly{s ;|f]tsf] cefj, lgb]{lzsfsf] cefj, ;dGjosf] cefj, la/fdL / pgsf k/Ljf/ klxrfg ug{ sl7g < s[kof o;sf] af/]df s]xL JofVof ul/lbg'x'G5 <

!)_ s]G›Lo lgsfoaf6 hgdfg;x?nfO{ ;dodf ;xL ;'rgf tyf ;]jfx? k|bfg ug{ ;'lglZrt ug{sf nflu s]-s:tf sfo{qmdx? ePsf 5g\ <

- ;'rgfsf] k|rf/ k|;f/ ug{sf] nflu ldl8of;Fu ;xsfo{ u/]sf]
- k|fOe]6 c:kftnx?;Fu zNos[of k|bfg ug{sf] nflu ;xsfo{ u/]sf]
- 8fS6/÷lrlsTzsx?nfO{ tflnd
- ;fdflhs :jf:Yo sfo{stf{x?nfO{, nlIft ju{sf] klxrfg ug]{ cEof; tyf tflnd

!!_ cGTodf, xfO{8«f];Ln zNos[ofsf] b/df ;'wf/ u/L ;a} nlIft hg;+Vofdf of] ;]jf k|bfg ug{ s'g} ;Nnfx jf ;'emfjx? lbg rfxg'x'G5 <

cGtjf{tf{sf] nflu cfˆgf] ;do lbg'ePsf]df tkfO{nfO{ w]/} wGojfb lbg rfxG5' . d}n] s]xL c? hfgsf/L kfpg k'gM ;Dks{ u/]df s'/fsfgL ug{ O{R5's x'g'x'G5 < tkfO{+nfO{ k'gM wGojfb .

**lhNnf hg:jf:Yo sfof{nosf xflQkfO{n] sfo{qmdsf] k|d'v JoQmL;Fusf] KII lgb{]lzsf**

lhNnf M

gu/kflnsf ÷ ufpFkflnsf M

ldtL M

sf]8 g+= M

pQ/bftfsf] ;fwf/0f hfgsf/L M

gfd M

kb M

;+:yfsf] gfd M

xfnsf] ;+:yfdf cg'ej M

xfnsf] kbdf cg'ej M

**kl/ro**

!_ s] tkfO{ dnfO{ tkfO{sf] lhNnfdf ;~rflnt lnDˆol6s lkmnfl/ol;; (xflQkfO{n]) sfo{qmdsf, d'Voto MMDP sfo{qmdsf] af/]df atfO{lbg'x'G5 <

@_ s] tkfO{ dnfO{ xfO{8«f];Ln zNos[of ;'ljwfsf] af/]df yk atfO{ lbg'x'G5 <

- zNos[ofsf] nflu tf]lsPsf c:ktfnx?
- 8fS6/ / :jf:Yo sd{rf/Lx?nfO{ tflnd / cled'lvs/0f
- ;|f]t-ah]6_ lgwf{/0f

#_ o;\ sfo{qmddf s]-s:tf ;'ljwfx? ;dfj]z 5g\ < k|f]a M oftfoft ;'ljwf , pkrf/sf] qmddf cfjf;sf] Joj:yf, x]/rfx ug]{nfO{ eQf, zNos[of kl5sf cGo ;'ljwfx?

$_ o; lhNnfdf xfO{8«f];Ln zNolqmof sfo{qmd ;'rf? ePsf] slt eof] <

%_ tkfO{;Fu clxn];Dd slt hgfn] o; ;'ljwf k|fKt u/] eGg] s'g} tYof+s 5 <

^_ o; lhNnfn] xfO{8«f];Ln zNolqmofsf] ;'ljwf k|fKt ug]{ ;+Vof j[l4 ug{ s]-s:tf /0fgLltx? agfPsf 5g\ <

&_ zNos[of ;]jfsf] nflu la/fdLnfO{ lhNnf c:ktfndf k|]if0f ul/g] sfo{ljwL s:tf] /x]sf] 5 <

*_ sfo{Gjogstf{ ePsf] gftfn] x]g{'kbf{, la/fdLnfO{ ;/sf/n] lgz'Ns k|bfg u/]sf] ;]jfsf] kx'+rdf k'Ug s]-s:tf afwfx? cfOk/]sf 5g\ < / o;nfO{ s] n] k|f]tzfxg\ ul//x]sf] 5 <

- plrt hfgsf/Lsf] k|rf/k|;f/ ug{sf] nflu IEC ;fdu|Lsf] sdL
- cGo la/fdLx?sf] pRr rfk
- ef}uf]lns cj:yfn] pkrf/df la/fdLnfO{ kg]{ afwfx?
- zSolqmofsf] nflu of]Uo la/fdLx?sf] klxrfgdf r'gf}tL

(_ ;]jfdf ;Demf}tf ug{'k/]sf s'g} To:tf If0fx? cfO{k/]sf 5g\ <

- ;|f]tsf] sdL
- lgb{]lzsfsf] sdL
- ;dGjosf] sdL

!)_ o; lhNnfn] hgdfg;x?nfO{ ;dodf ;xL ;'rgf tyf ;]jfx? k|fbfg ug{sf nflu yk s]-s:tf sfo{qmdx? ;~rfngdf NofPsf 5g\ <

!!_ cGTodf, xfO{8«f];Ln zNos[ofsf] b/df ;'wf/ u/L ;a} nlIft hg;+Vofdf of] ;]jf k|bfg ug{ s'g} ;Nnfx jf ;'emfjx? lbg rfxg'x'G5 <

**lhNnf c:ktfn xfO{8«f];Ln zNos[of ;DalGwt** **JoQmL;Fusf] KII lgb{]lzsf**

lhNnf M

gu/kflnsf ÷ ufpFkflnsf M

c:ktfnsf] gfd M

ldlt M

sf]8 g+= M

pQ/bftfsf] ;fwf/0f hfgsf/L M

gfd M

kb M

;+:yfsf] gfd M

xfnsf] ;+:yfdf cg'ej M

xfnsf] kbdf cg'ej M

**kl/ro**

!_ xfO{8«f];Lnsf la/fdLx?nfO{ k|bfg ul/g] ;]jfx? s]-s] x'g\ atfO{ lbg'x'G5 <

@_ o; c:ktfnn] xfO{8«f];Lnsf la/fdLx?nfO{ zNolqmof ;]jf k|bfg ug{ yfn]sf] slt eof] <

#_ o; sfo{qmddf s]-s:tf ;'ljwfx? ;dfj]z 5g\ < k|f]a M oftfoft ;'ljwf , pkrf/sf] qmddf cfjf;sf] Joj:yf, x]/rfx ug]{nfO{ eQf, zNos[of kl5sf cGo ;'ljwfx?

$_ tkfO{;Fu clxn];Dd c:ktfnaf6 slt hgfn] o; ;'ljwf k|fKt u/] eGg] s'g} tYof+s 5 <

%_ o; c:ktfndf zNos[of ;]jfsf] nflu la/fdLnfO{ k|]if0f ul/g] sfo{ljwL s:tf] /x]sf] 5 <

- :yfgLo :jf:Yo ;+:yfx?n] k|]if0f u/]sf] kq (Referral slip)
- LF sf] sf/0fn] g} xfO{8«f];Ln ePsf] eg]/ lglZrt ul/Psf] k|df0f

^_ sfo{Gjogstf{ ePsf] gftfn] x]g{'kbf{, la/fdLnfO{ ;/sf/n] lgz'Ns k|bfg u/]sf] ;]jfsf] kxF'rdf s]-s:tf afwfx? cfOk/]sf 5g\ < / o;nfO{ s]n] k|f]Tzfxg\ ul//x]sf] 5 <

- zNolqmof ;fdu|Lx?sf] pknAwtf gePsfn]
- k/fdz{sf] nflu IEC ;fdu|Lsf] sdL
- cGo la/fdLx?sf] pRr rfk
- tflnd k|fKt 8fS6/x?sf] sdL, ;/sf/af6 tflnd k|bfg gul/Psf]
- zNos[of kZrftsf] Joj:yfkgdf r'gf}tL
- zNos[ofsf] nflu la/fdLx?sf] klxrfgdf r'gf}tL
- la/fdLnfO{ c:ktfn;Dd cfO{k'Ug ef}uf]lns afwfx?

&_ xfO{8«f];Ln zNolqmof ;]jf ;~rfng kZrft s'g} sf/0fj; ;]jf k|jfxdf ;Demf}tf ug{'k/]sf s'g} To:tf If0fx? cfO{k/]sf 5g\ <

- ;|f]t, lgb]{lzsf / ;dGjodf sdL
- tflnd k|fKt 8fS6/sf] sdL, l/St b/aGbL, 8fS6/x?nfO{ cfly{s k|f]T;fxgsf] sdL

*_ o; c:ktfnn] hgdfg;x?nfO{ ;dodf ;xL ;'rgf tyf ;]jfx? k|bfg ug{sf] nflu yk s]-s:tf sfo{qmdx? ;+rfngdf NofPsf 5g\ <

- ;"rgf k|rf/k|;f/ ug{sf] nflu ldl8of ;Fusf] ;xsfo{
- lghL c:ktfnx? ;Fusf] ;xsfo{
- :yfgLo :jf:Yo sd{rf/Lx?nfO{ tflnd k|bfg, la/fdL klxrfg / k|]if0f ug]{

(_ cGTodf, xfO{8«f];Ln zNos[ofsf] b/df ;'wf/ u/L ;a} nlIft hg;+Vofdf of] ;]jf k|bfg ug{ s'g} ;Nnfx jf ;'emfjx? lbg rfxg'x'G5 <

**;d'bfosf dlxnf :jf:Yo :jo+;]jLsfx?;+usf] FGD** **lgb{]lzsf**

5nkmn ;+rfnsn] ;a} ;xefuLx?nfO{ :jfut u/L kl/roaf6 sfo{qmd ;+rfng ug]{5 .

lhNnf M

gu/kflnsf ÷ ufpFkflnsf M

j8f g+= M

ldlt M

sf]8 g+= M

o; 5nkmndf tkfO{x? ;a}nfO{ :jfut 5 / ;xefuL x'g ;xdt x'g'ePsf]df wGoafb 5 . tkfO{Fx?sf] ljrf/ dxTjk"0f{ x'g] ePsfn] tkfO{x?nfO{ ;xefuL x'g cg'/f]w u/]sf xf}F . o; 5nkmn sl/a cfwf 306fsf] x'g]5 . olb ;xdlt hgfpg'ePdf, tkfO{x?n] af]n]sf s'/fx?sf] /fd|f];Fu hfgsf/L kfpg d o; 5nkmnnfO{ /]s8{ ug{ rfxG5' . oBlk, tkfO{x?n] lbg'ePsf hfgsf/Lx? / o; 5nkmnsf s'/fx? uf]Ko /flvg]5 ;fy} cWoogsf] k|of]hgsf] nflu dfq k|of]u ul/g]5

**5nkmn sfo{qmdsf lgodx? M**

- s[kof ;xL / ;fFrf] hfgsf/Lx? lbg'xf]nf .
- Ps-Ps ub}{ af]lnlbg'xf]nf . ;a}hgf 5nkmndf ;xefuL eO{lbg'xf]nf . cfˆgf] s'/f /fVg] s'g} qmd /x]sf] 5}g t/ cfˆgf] ;fyLn] af]ln;s]kl5 dfq cfˆgf] egfO{ /fVg'xf]nf .
- oxfF s'g} ;xL jf unt pQ/ x'g]5}g . tkfO{Fx?sf] cfˆg} ljrf/x? x'G5g\, cGo sf]xLn] To; ljrf/df ;xdt jf c;Gt':6L hgfpg h?/L 5}g .

!_ tkfO{sf] If]qdf xfO{8«f];Ln ePsf JolQmx?sf] ;+Vofsf] 5f]6s/Ldf hfgsf/L k|bfg ug{ ;Sg'x'G5 < -s'n ;+Vof, pkrf/ gkfPsfsf] ;+Vof _

@_ ;/sf/n] xfO{8«f];Lndf k|bfg ug]{ pkrf/ ;'ljwfsf] af/]df tkfO{nfO{ s] yfxf 5 <

#_ tkfO{x?n] :jf:Yo ;+:yfaf6 xfO{8«f];Ln ;DaGwL la/fdL klxrfg ug]{, k/fdz{ lbg] / k|]if0f ug]{ s'g} k|sf/sf] tflnd jf cled'lvs/0f kfpg' ePsf] 5 < k|f]a M tflnd s:tf] lyof] <

$_ tkfO{n] la/fdLnfO{ c:ktfndf pkrf/ ug{ hfgsf] nflu k/fdz{ lbg'ePsf] jf k|]if0f ug{' ePsf] 5 < k|f]a M tkfO{ la/fdLnfO{ s;/L ;Demfpg'x'G5, slQsf] ;Demfpg'x'G5, slQsf] ;kmn x'g'ePsf] 5 <

%_ ;fwf/0ftof, xfO{8«f];Ln tyf xfO{8«f];Ln ePsf JolQmdf ;fdflhs tyf ;fF:s[lts b[:6Lsf]0f s:tf] kfpg' ePsf]5 <

- la/fdLnfO{ ;fdflhs jf kfl/jfl/s ?kdf ul/g] Jojxf/, ;fdflhs lqmofsnfkdf ;xefuL x'g glbg], gk'+u;stf, cGo JolQmdf /f]u ;g]{ eo, kl/jf/n] pk]Iff ug]{, la/fdLsf] j}jflxs ;DaGwdf ;d:ofx? pTkGg x'g],
- k/Dk/fut pkrf/sf dfWod h:t} wfdL, emfFqmLx?sf]df hfg'
- k"j{ hGdsf] s'sd{n] ubf{ eujfgn] >fk lbPsf] h:tf s'/fdf ljZjf; ug{' , j+zf0f'ut /f]u ePsf]df ljZjf; ug'{

^_ tkfO{x?sf] ljrf/df la/fdLnfO{ ;]jfsf] kx'+rdf k'Ug s]-s:tf afwfx? cfO{k/]sf 5g\ < / o;nfO{ s]n] k|f]T:ffxg ul//x]sf] 5 <

- kfl/jfl/s ;xfotfsf] sdL, la/fdLn] v'n]/ cfˆgf] /f]usf] af/]df s'/f ug{ grfxg] , /f]u kQf nufpg n}lËs ;d:ofx? cfOkg]{, la/fdL jf a/fdLsf] kl/jf/nfO{ nfhsf] cg'ej x'g]
- la/fdLnfO{ kl/jf/sf] af]emsf] ?kdf x]l/g]
- ;]jfsf] kx'+rdf ef}uf]lns afwf cfO{k/]sf]
- cfly{s cj:yf
- zNolqmof kZrft gk'+;s\ x'g] ;fdflhs nf~rgf
- /f]usf] pkrf/ g} x'g g;Sg] ljZjf;, zNolqmofdf d[To' x'g] eo

&_ tkfO{sf] ljrf/df xfO{8«f];Ln ePsf JolQmx?df k|efjsf/L ;]jf k|bfg ug{ s] ul/g'k5{ <

- ;fd'bflos ;r]tgf sfo{qmd
- oftfoft vr{ k|bfg u/L k|f]T;fxg ul/g'kg]{
- k'gM:yfkgf ;]jf k|bfg ul/g'kg]{

*_ tkfO{sf] ljrf/df tkfO{n] xfO{8«f];Ln ePsf JolQmx?nfO{ :jfYo ;+:yfx?df k|]if0f u/L cfjZos :jf:Yo pkrf/ k|fKt ug{df s;/L ;xof]u ug{ ;Sg'x'G5 < tkfOsf] e"ldsf s] x'g;S5 <

- k/fdz{ / k|]if0f;DalGw tflnd, la/fdLnfO{ k|]if0f u/]afkt tkfO{nfO{ cfly{s k|f]T;fxg
