# Supplementary material for: Exploring determinants of hydrocele surgery coverage related to Lymphatic Filariasis in Nepal: An implementation research study
Source: PLoS One. 2021 Feb 26;16(2):e0244664. doi: 10.1371/journal.pone.0244664 (PMC7909642; doi:10.1371/journal.pone.0244664)
Supplement: S3 File — (DOCX) [file pone.0244664.s003.docx]

| **Stakeholder from Central Level (KII)** | |
| --- | --- |
| Program implementation strategy | Hydrocele status |
| - MDA, MMDP - Health workers, volunteers training - MMDP advocated since 2003, Hydrocele surgery since 2016 - Surgery available in all govt. hospital - Financial support to all govt. hospital - Nepal government, WHO and Liverpool school of tropical medicine major donors for hydrocele surgery camp - Package cost per patient, gradual increase of support - 7327 beneficiaries till date out of expected beneficiary of 9000-9500 - FCHVs notify cases, health workers verify cases - Information dissemination to all health facilities - Budget allocated for information dissemination - FCHVs, health workers aware of hydrocele surgery camp - Press conference, news coverage through national TV, radio prior to surgery camp - Extension of elimination target beyond 2020 since elimination is not possible - Morbidity management/hydrocele surgery to be continued post LF elimination | - Providing only free surgery more than enough, patients are self-motivated and self-oriented for overcoming shame - No demand of transportation facilities - Not witnessed discrimination towards hydrocele patient - Not associated with mortality - Self-discrimination by patient |

| **Stakeholders from District Level (Kanchanpur and Dhading), KII** | |  |
| --- | --- | --- |
| Program implementation strategy | Hydrocele status | Recommendation |
| - Budget disseminated by central level - Free surgery including treatment for any post-surgery complication - Executive committee formed after receiving budget for organizing camp - Screening prior to camp - Workshop and planning meeting with stakeholder - Free surgery, medicine, lab tests, post-operative care - Incentive for surgery team - Package of 6000 rupees - Insufficient budget for information dissemination - Most time consumed in planning, hiring surgeon and other health workers and negotiating their fee/charge - Hiring surgeon lengthy process - Free surgery only during camp, otherwise surgery not possible in the district hospital - Not fixed plan until budget disseminated - Review meeting once a year from central level | - Surgery beneficiaries target not achieved - Camp organized in rice plantation season - Patients don’t want to risk surgery in plantation season - Many cases of hydrocele in Dhading - Delay in case diagnosis - Lack of awareness - Sub-optimal information dissemination - Less focus on preventive aspect - Fear of surgery among general public and patient | - Provide list of surgeon from central level - More focus on conducting camp and less on awareness raising - Winter season favourable for surgery - Conduct mobile camp in remote, inaccessible place - Awareness education through school - Proactive involvement of District health office - Program certainty and provide free hydrocele surgery as mainstream service all the year around - More focus and incentive to poor people - Effective information dissemination |

**FGD with Female Community Health Volunteers (Kanchanpur and Dhading)**

| Hydrocele Status | Knowledge and Perceptions | Discrimination/Stigmatization | Recommendation |
| --- | --- | --- | --- |
| - Old and poor people seem to be affected mostly - Recurring hydrocele - Rich people get treatment in private hospital in the cities, so no cases seen in people of good economic background - Have knowledge of surgery - Poor people cannot afford to not work and rest after getting surgery - Ineffective information dissemination - Deny having hydrocele - Offended when confronted - No pain observed - Loss of daily income during and after surgery - No emergency and life threatening | - Fear of complication after surgery - Avoid certain foods such as tomato, potato - Avoid cold and rain - Hydrocele attributed to col, strenuous physical work - No significant pain and suffering - Home remedies to reduce swelling and pain - Warm with pre-heated bricks/stone - Hydrocele as weakness - Associated with sexual disease - Belief that post-surgery needs lots of rest and nutrition to gain - Perceived weakness after surgery - Scared of surgery | - Self-stigmatized - Self-discrimination - Don’t want to talk about it - Hesitation to talk with female - Family members do not hide - No societal discrimination - Patient often looked down upon - Prefer treatment with medicine than surgery - Prefer private hospital | - Mobile camps in remote places - Counselling training for FCHVs - More information on hydrocele causation and treatment-Lack of trust towards FCHVs due to poor knowledge of hydrocele - Regular and frequent camps - Transportation allowance and other incentive |

**Respondents Interview (IDIs, Kanchanpur and Dhading)**

| **Personal Challenges** | | |
| --- | --- | --- |
| Patients undergone surgery | Patients with no surgery | Family of patient |
| - Teasing and name calling - Painful and uncomfortable - Avoid religious ceremonies and family gatherings - Confined at home - Embarrassment in front of sisters and family - Low self-esteem, ashamed of having hydrocele - Self-conscious - Clothing restrictions due to pain and appearance - Conceal having hydrocele during marriage - Societal scrutiny - Trouble in walking, moving restrictions - Unable to work - Difficulty in doing day to day activities - Scared of surgery - Fear of not being able to produce children - Bad karma | - Difficulty in peeing - No pain and don’t feel uncomfortable - Pain around the waist - Shame/embarrassment - Old age - Put-off surgery for saving money for having surgery - Scared of surgery - Trouble in walking | - No pain and not uncomfortable - Pain and difficulty in urination - Difficulty to work |

| **Knowledge and Perception** | | |
| --- | --- | --- |
| Patient undergone surgery | Patient with no surgery | Family member of patient |
| - Gradual increase in size with age - Have asthma - Associated with cold - Associated with heavy physical work/strain like driving, riding bicycle - No knowledge of cause - Scared of surgery - Knowledge of surgery as treatment - Mosquito bite | - Gradual increase in size with age - Have diabetes - Size increase in summer, hot weather - Doesn’t hurt, why bother to have surgery - Back pain - Associated with trauma, accident - No knowledge of cause - Scared of losing scrotum, of death - Recurring hydrocele - Weakness, leg cramping | - Associated with injury - Recurring hydrocele - No knowledge of cause - Gradual swelling - Mosquito bite - Not life threatening - Cannot be treated completely |

| **Patients coping mechanism and home remedies** | | |
| --- | --- | --- |
| Patient undergone surgery | Patient with no surgery | Family member of patient |
| - Eat parrot meat, rub crow’s burnt foreskin - Apply cow’s butter - Herbal Medicines - Rub nettle leaves soaked after soaking in water - Ineffective home remedies | - Tightfitting clothes, double underwear for hiding hydrocele - Medicine for pain relief - Drink salt and water (ORS) - Avoid eating potato and meat - Warm the swollen area with hot ash wrapped in cloth - Herbal medicine(rub oiled leaf with, warm in heat and rubbing) | - Apply crow’s blood on the swollen area - Not disclosing to wife and family member - Avoid eating meat, fish - Rubbing with Imli (kind of herbal plant) leaves and melted butter |

| **Barriers** | | | |
| --- | --- | --- | --- |
| Stakeholder and FCHVs | Patients undergone surgery | Patient without surgery | Family members of patients |
| - Incomplete MMDP mapping in all endemic districts - Lack of awareness among patients - Lengthy process of treatment - Lack of surgeon and infrastructures in government hospitals - Change in federal structure - Power collision between district health office and district hospital due to unclear roles and responsibilities - Many vacant and unfulfilled post in district hospitals - Episode, one-time event - Mistrust in govt. services and doctors - Expensive for poor people, with transportation and accommodation and food cost combined for at least 3-4 days, not only patient cost but also for care-taker coming along - Service available only in the district hospital in district headquarter, which makes it difficult for people residing far away to come and receive surgery - Retention of skilled and qualified human resource in govt. hospital, resulting in constant vacant positions - Late budget dissemination from central level - More time consumed in hiring consultant and at planning stage, resulting in haste execution of surgery camp - Difficulty in coordination between various stakeholders in the district - Lack of proper information dissemination - Lack of trust in govt. hospital from general public - Uncertainty of program and budget dissemination - Lack of coordination between district health office and district hospital-hospital as a sole entity for carrying out hydrocele surgery - Recent change in federal structure in the country which resulted in district health office and district hospital under different ministries | - No information about free surgery facility - Lack of money - Long queue in govt. hospital - Lack of surgeon in govt. hospital - Mistrust in govt. services and doctors - Lack of money - Complication after surgery - Avoid risk in govt. hospital - old age, carelessness - Shame/embarrassment of coming out and accepting of having hydrocoele - Scared of being impotent after surgery | - No information about free surgery facility - Lack of money - Difficulty in accessibility - Mistrust in govt. services and doctors - Lack of surgeon in govt. hospital - Lack of money - Disrespect and bad treatment by health workers in govt hospital - Lack of surgery availability while gone for treatment - Scared of death after surgery - Surgery considered critical, fear of losing income during resting period, post-surgery weakness | - Lack of doctor and facility in govt. hospital - Belief in home remedies - No information about free hydrocele surgery - Afraid of losing job after surgery - Saving up money for having surgery - Old age - Scared of surgery - Asthma - Mistrust in government services - Frustrations with govt. service/health workers - Hard to convince the husband |

| **Recommendations** | | |
| --- | --- | --- |
| Patients undergone surgery | Patient without surgery | Family members of patients |
| - Prompt service, proper counselling - Proper dissemination of service - Competent Doctors availability - Accessibility - Priority for poor people - Travel allowance - Proper information on total cost, treatment procedure, duration - Information about hydrocele surgery camp - Awareness raising activities in communities | - Prompt service, proper counselling - Proper dissemination of service - Competent doctors availability - Accessibility - Priority for poor people - Travel allowance - Proper information on total cost, treatment procedure, duration - Information about hydrocele surgery camp - Awareness raising activities in communities - Surgery preferable in winter - Privacy and male doctors | - Free and prompt service - Respect towards patient by health care workers - Information about free hydrocele surgery through neighbours/friends and public announcement |

**Enablers:**

| Stakeholders/FCHVs/Patients |
| --- |
| - Outsourcing and coordination with private hospitals - Commitment and accountability from stakeholders at all level - Improvement in budget channel - Change in federal structure - Scaling-up of MMDP mapping in rest of the endemic districts - Commitment from the donors and stakeholders - Very happy and comfortable after surgery - Confident and energetic after surgery - Regret not doing surgery sooner - Regular and increase in income - Information regarding the camp through radio, FCHVs, local health facilities |
