# Supplementary material for: Exploring determinants of hydrocele surgery coverage related to Lymphatic Filariasis in Nepal: An implementation research study
Source: PLoS One. 2021 Feb 26;16(2):e0244664. doi: 10.1371/journal.pone.0244664 (PMC7909642; doi:10.1371/journal.pone.0244664)
Supplement: Attachment — Submitted filename: Response to Reviewers.docx [file pone.0244664.s005.docx]

September 30, 2020

Re: Resubmission of manuscript, “Exploring determinants of hydrocoele surgery coverage related to Lymphatic Filariasis in Kanchanpur and Dhading districts of Nepal: An implementation research”, PONE-D-20-21081

Yaobi Zhang, M.D., Ph.D.

Academic Editor

PLOS ONE

Dear Dr. Zhang,

Thank you for inviting us to submit a revised draft of our manuscript titled, “Exploring determinants of hydrocoele surgery coverage related to Lymphatic Filariasis in Kanchanpur and Dhading districts of Nepal: An implementation research” to PLOS ONE. We really appreciate the time and effort you and each of the reviewers have dedicated by providing insightful feedback to improve our paper. We have incorporated changes that reflect the suggestions you have graciously provided. We hope that the responses we have provided, and the changes made satisfactorily address all the concerns you and the reviewers have noted.

To facilitate your review of our revisions, point-by-point responses to the comments are provided below.

**ACADEMIC EDITOR COMMENTS:**

1. Please ensure that your manuscript meets PLOS ONE's style requirements, including those for file naming. The PLOS ONE style templates can be found at

<https://journals.plos.org/plosone/s/file?id=wjVg/PLOSOne_formatting_sample_main_body.pdf> and

<https://journals.plos.org/plosone/s/file?id=ba62/PLOSOne_formatting_sample_title_authors_affiliations.pdf>

- **Response:** Thank you very much for the suggestions. We have followed the requirements.

2. In your Methods section, please provide additional information about the demographic details of your participants. Please ensure you have provided sufficient details to replicate the analyses such as: a)  a description of any inclusion/exclusion criteria that were applied to participant inclusion in the analysis and b) a table of relevant demographic details.

- **Response:** Thank you for your suggestions. We agree with you and have incorporated this in the method section under the sub-head “Sampling and sample size” (page-9, Line208-210). A table depicting a sociodemographic profile has been added in the result section (page-12,13).

3. Please include additional information regarding the interview guide used in the study and ensure that you have provided sufficient details that others could replicate the analyses. For instance, if you developed a guide as part of this study and it is not under a copyright more restrictive than CC-BY, please include a copy, in both the original language and English, as Supporting Information. In addition, please include further details of the development and validation of this tool.

- **Response:** Thank you for your insight. We have uploaded the interview guides as Supporting Information. Details are provided under sub-head “Data collection and research instruments” ( page-9, Line 218-224).

4. Please ensure you have thoroughly discussed any potential limitations of this study within the Discussion section, including the potential introduction of biases during data collection and sampling.

- **Response:** Thank you for raising an important issue. We have addressed this in our discussion section (page-31, final paragraph)

5. Please modify the title to ensure that it is meeting PLOS’ guidelines (<https://journals.plos.org/plosone/s/submission-guidelines#loc-title>). In particular, the title should be "specific, descriptive, concise, and comprehensible to readers outside the field" and in this case we have concerns that the title is long and contains errors of grammar. An alternative title suggestion is: "Exploring determinants of hydrocoele surgery coverage related to Lymphatic Filariasis in Nepal: An implementation research study".

- **Response:** Thank you for your suggestion. We have incorporated your suggestion in the revised manuscript.

6. We note that you have indicated that data from this study are available upon request. PLOS only allows data to be available upon request if there are legal or ethical restrictions on sharing data publicly. For information on unacceptable data access restrictions, please see <http://journals.plos.org/plosone/s/data-availability#loc-unacceptable-data-access-restrictions>.

In your revised cover letter, please address the following prompts:

a) If there are ethical or legal restrictions on sharing a de-identified data set, please explain them in detail (e.g., data contain potentially identifying or sensitive patient information) and who has imposed them (e.g., an ethics committee). Please also provide contact information for a data access committee, ethics committee, or other institutional body to which data requests may be sent.

b) If there are no restrictions, please upload the minimal anonymized data set necessary to replicate your study findings as either Supporting Information files or to a stable, public repository and provide us with the relevant URLs, DOIs, or accession numbers. Please see <http://www.bmj.com/content/340/bmj.c181.long> for guidelines on how to de-identify and prepare clinical data for publication. For a list of acceptable repositories, please see <http://journals.plos.org/plosone/s/data-availability#loc-recommended-repositories>.

We will update your Data Availability statement on your behalf to reflect the information you provide.

- **Response:** Thank you for your assessment. We have made de-identified and categorised data sets available as Supporting Information and other relevant data are within the paper. However, the raw transcripts generated cannot be made publicly available for ethical reasons. Public availability would compromise participants confidentiality as it contains personal information of the participants as well as references throughout most of the transcripts such as job title, location, which has the potential to identify the respondents. Our consent form explicitly states that the no information would be made available that could compromise their identity. Our institution does not have an established point of contact to field external request for access to raw data. Hence, additional relevant information can be made available on reasonable request to the corresponding author. We hope the presented data sets meets the requirement of the journal.

7. Please include captions for your Supporting Information files at the end of your manuscript, and update any in-text citations to match accordingly. Please see our Supporting Information guidelines for more information: http://journals.plos.org/plosone/s/supporting-information.

- **Response:** Thank you for your suggestion. We have incorporated accordingly.

**REVIEWER 1 COMMENTS:**

1. The manuscript has the potential to make a critical contribution to published literature about barriers to access to hydrocele surgery. However, it needs some significant rewriting to link the findings to previous studies, clearly explain the methodology used, and clarify the data analysis conducted. The results also could benefit from inclusion of some quantitative statistics, e.g. of the 10 people interviewed, 7 knew the cause of hydrocele. Finally, the manuscript could benefit from an English-language editor; I did not include specific editorial suggestions in my comments below.
   Specifically,
   - The introduction section needs reworking and editing. For example, in some parts (e.g., lines 105-114) it reads like an advocacy document as opposed to a research article. As another example, referring to the LF Elimination Program consistently throughout would be useful; instead it is referred to at various times as the MOHP, EDCD, or the LF program.

- **Response:** Thank you very much for providing these insights. We agree with your assessment and have made significant changes in the introduction, methodology as well as result section. A table has been added to highlight the socio-demographic data quantitatively. The final copy of the manuscript has been edited by an English-language editor.

2. Lines 89-90. Please clarify what you mean by ‘scale up the MMDP component of the LF elimination program’ if MMDP services are already available in all endemic areas. This seems contradictory.

- **Response:** Thank you for your assessment. We agree that the sentence seemed rather contradictory. Thus we have explained further by giving a detailed description in page no. 5-6, line 112-124.

3. Line 110. Please include references to past research on hydrocele surgery access here, as well as throughout the introduction. The discussion section has a few references, but more exist on hydrocele surgery barriers as well as access to essential surgery in general and would be useful to cite.

- **Response:** Thank you for your suggestion. We have incorporated more references.

4. The background section would benefit from including the elements on MMDP needed for WHO to validate the elimination of LF as a public health problem and how this research links to those elements/helps national LF elimination programs overcome barriers to elimination

- **Response:** Thank you for your assessment. We have incorporated your suggestion by giving a little background on WHO’s guideline on MMDP as well as national elimination program. It is reflected in page no. 4 (line 89-95) and page no. 5 (line 111-114).

5. Line 114. The study seemed to aimed to understand the barriers to surgical access as opposed to addressing them. Consider rewording. It also would be useful to clarify here or in the discussion what was to be done with this increased understanding – make policy recommendations to the MOHP? Share with global policymakers?

- **Response:** Thank you for your valid assessment. We have incorporated your suggestions. (page 7, line 151-154) and addressed more in the discussion section.

6. Throughout the methods section, it would be useful to standardize presentation of details about in-depth interviews (IDI), key informant interviews (KII) and focus group discussions (FGD) and discuss in the same order in each sub-section. As written, it was slightly unclear who was included in each group, what the sampling methods were for each, what data analysis was done for each, etc. This would also hold true for presentation of the results – a consistent approach could help readers comprehend what was learned from each group of respondents.

- **Response:** We agree with your assessment and have incorporated changes throughout the methods and result section.

7. Why were the three family members included in the IDIs? To me, it is more interesting to see what responses comes from the patients vs all others and inclusion of the family members with the patients confuses the perspective of the patients.

- **Response:** You have raised several interesting questions. In the beginning of the study, we also discussed whether including family members would be necessary or not. After discussion, we decided to include family members of hydrocoele patients who have not had undergone surgery yet, hoping that it would provide us some in-depth information on what the family or community thinks about hydrocoele and underlying reasons for not undergoing surgery (yet) in case hydrocoele patient would not disclose it openly.

8. Line 138-140. Although mentioned that purposive sampling ensures diversity in terms of age, SES, urban/rural, the text states that the purposive selection was only based on in terms of highest number of hydrocele patients. Consider deleting 139-140. It is also unclear when snowball sampling was employed.

- **Response**: Thank you for this insight. We have deleted the sentence. Some hydrocoele patients were identified with the help of those who participated in the interview. Hence both purposive and snowball sampling were used to identify IDI respondents.

9. Line 143: Related to this, in the results, clearly state number (%) of patients located and number of patients consenting to take part.

- **Response:** Thank you for the suggestion. We have incorporated in the result section.

10. Line 147: how many FCHVs took part in each FGD?

- **Response:** Seven FCHVs in Kanchanpur and 5 FCHVs in Dhading took part in FGDs.

11. Line 148: What was the purpose of the telephone inquiries prior to the interviews?

- **Response:** Telephone inquiries were done primarily to inquire about their availability as well as confirm their consent to participate.

12. Line 156: How was data triangulation done? By whom?

- **Response:** Data triangulation was done by cross-checking data from different group of respondents, by the corresponding author.

13. Line 168: How was data saturation measured?

- **Response:** Data saturation was insured by making sure all the questions and variables have been covered from all group of respondents and no new information was gathered.

14. Lines 176-177: Who transcribed the data? Who ensured data quality?

- **Response:** Data was transcribed by principal interviewer (research assistant and corresponding author). Data quality was ensured by the principal interviewer and corresponding author.

15. Lines 179-184: This is very vague. Was any specific qualitative data analysis guidance followed to code and group the data? Did more than one person code the data? How was intercoder variance addressed?

- **Response**: Thank you for your insights. Data coding and analysis were done manually by the corresponding author.

16. Lines 197-206: It could be helpful to organize this by IDI, KII, and FGD sociodemographic characteristics and include IDI median age (range), rate of refusal, # of years with hydrocele, occupation, # had surgery at govt camp vs private hospital; KII sex, age, # years in current position; and FGD age, # years in the position.

- **Response**: Thank you for the suggestion. We have incorporated your feedback by adding a table depicting the socio-demographic profile of the respondents.

17. Lines 278-280: As described, that is more budget than most LF programs allocate for hydrocele camps or routine hydrocele services. Might be useful to include some information about what activities the respondent felt were lacking, if that is available.

- **Response:** We agree with your observation. In our revisions, we have attempted to address this more clearly and we think that the barrier section also highlights your suggestion, We hope that you agree (line 413 and 424).

18. The entire barriers section might flow better if it were organized by the steps in accessing services, e.g. first a patient needs to recognize they need surgery (hydrocele manifestation), second their fear of surgery needs to be overcome, then their mistrust of government services needs to be overcome, then they need to be aware of the camps, and finally economic barriers need to be overcome.

- **Response:** Thank you for your important insight. We have incorporated your suggestions accordingly in our revision.

19. The discussion section might flow better if knowledge and perception of hydrocele comes before the knowledge and perception of the free hydrocele surgery program.

- **Response:** Thank you for your insightful observation. We have incorporated it accordingly.

20. How were results shared with the MOHP and others responsible for improving services?

- **Response:** The stakeholders were briefed about the preliminary findings during the time of KII. Detail findings were shared later on after completion of report writing online due to COVID-19 restrictions.

21. What specific policy or other recommendations do the authors have to the MOHP and/or the LF elimination program to overcome these barriers?

- **Response:** We have reflected on this comment by incorporating arguments and recommendations related to our findings as well as other studies throughout the discussion section.

22. What recommendations do the authors have for other countries implementing similar programs?

- **Response:** You have asked a very interesting question. We believe that contextual difference should be addressed by policy makers and stakeholders alike wherever this program is being implemented and tailored according to the ground reality instead of blanket approach. In fact, within countries also, approaches could or should be varied and flexible to cultural contexts.

**REVIEWER 2 COMMENTS:**

The manuscript by Yonzon et al. aims to identify barriers and enablers of access to surgical services for LF-related hydrocoele in Nepal. Data for the study come from a series of qualitative surveys among hydrocele patients, family members, community health volunteers and other stakeholders from two high-burden districts of the country. This is an important study, as there is currently limited information in the literature on this topic, and the authors do a good job of placing this study within the wider context of the global lymphatic filariasis elimination effort and morbidity service provision. While the conclusions are supported by the data, the very small sample size of the hydrocele patients for the study (only 5 people who underwent hydrocele surgery and 4 hydrocele patients who had not) severely weakens the study. It does not invalidate the data obtained from the study participants and other study participant groups, but it does call into question the study design and representativeness of the core findings—particularly as these participants were spread over two districts.

Specific comments are provided below.

**Major comments:**

1. Sample size/study design: Why were so few hydrocele patients included in this study? How was snowball sampling employed if the numbers of participants in each survey type was so small?

- **Response:** Thank you for your important insights. We agree that the study employed a very small number of respondents and have thus acknowledged it as one of the limitations to our study. While collecting data, after making sure that all the variables were collected as per the interview guide, and no new information was obtained from the respondents, we felt it that it had reached saturation. Some hydrocoele patients were identified with the help of those who participated in the interview. Hence both purposive and snowball sampling were used to identify IDI respondents.

2. The Results section is difficult to follow as the authors organize results thematically rather than by sample group (KII, FGD, IDI). The result is repeated switching between sample groups that disrupts the logical flow of the Results section. If possible, I recommend re-organizing results by sample group (KII, FGD, IDI) then highlighting specific themes within each group. If this is not feasible, then at least clarify in each paragraph which survey group is being described.

- **Response:** Thank you for your suggestion. We believe this is a very valid assessment and have tried to incorporate this suggestion throughout result section making sure that the results are not repeated and the flow is maintained.

3. I recommend a table summarizing, by survey group, the socio-demographic characteristics and quantitative analysis of key questions (e.g. the proportion of respondents who know the cause of hydrocele; the proportion who know that it is transmitted by mosquito, etc.).

- **Response:** Thank you for your suggestions. We have added a table highlighting the socio-demographic profile of the respondents (page 12, 13)

4. Did the study ask hydrocele patients about enablers to seeking surgery? I don’t see such data reported

- **Response:** Thank you very much for an important query. While defining enablers, we tried to focus on the factors which enabled the surgery (camp) to take place in general programmatic sense and thus identifying the barriers would highlight the issues that is needed to be addressed in addition to the already available conditions which made hydrocoele surgery possible in the first place. This is presuming that understanding and then addressing the identified barrier (patient side as well as programmatic side), would automatically enable the patient to access the service.

5. The Discussion should include a section acknowledge the limitations of the study—including, but not limited to the study’s small sample size, age bias in sample population, geographic bias, etc

- **Response:** Thank you for your important insight. We have incorporated your suggestion and made changes (page 31, line 718-728).

6. I appreciate that English likely is not the authors’ first language. However, the manuscript would benefit from editorial assistance to improve grammar and overall English.

- **Response**: We agree with you. Final manuscript copy has been edited by an English-language editor.

**Minor comments:**

7. Introduction, 3rd paragraph: Suggest adding “in Nepal” to the sentence beginning on line 95 to clarify the paragraph’s contents relate to Nepal specifically.

- **Response:** Thank you for your suggestion. We have revised the introduction section and have incorporated your suggestion. We hope you agree that these changes are better.

8. Methods (line 130 and 133): please verify the numbers “1,71,304” and “3,36,067”

- **Response:** Thank you for your important insight. We have made corrections.

9. Methods. I don’t see that “stakeholders” is defined. Who are they?

- **Response**: Thank you for important observation. We have clarified in the method section sub-head “research type and design” (page 7, line 167-168).

10. Methods: Research setting. Please add information on how many surgical camps have been conducted in the survey districts.

- **Response:** Thank you for your suggestion. We have incorporated accordingly (page 8, line 184-187)

11. Please clarify which sample population is referred to in the first line of the results (line 197)

- **Response:** Thank you for your insight. We have clarified that (page-11, line 262)

12. Please double check the author listing for reference #12 – it does not look correct.

- **Response:** We agree with you and re-checked the author listing. It might be due to the name of one of the author (deSilva), we believe it is the correct listing as the author’s name is Nilanthi R. deSilva.

Thank you once again for giving us the opportunity to strengthen our manuscript with your valuable comments and suggestions. We have worked hard to incorporate your feedback and hope that these revisions persuade you to accept our submission.

Sincerely,

Choden Lama Yonzon

Corresponding Author

Universitas Gadjah Mada

Yogyakarta, Indonesia

[yonzon.chhoden.cy@gmail.com](mailto:yonzon.chhoden.cy@gmail.com)
