# Supplementary material for: Exploring determinants of hydrocele surgery coverage related to Lymphatic Filariasis in Nepal: An implementation research study
Source: PLoS One. 2021 Feb 26;16(2):e0244664. doi: 10.1371/journal.pone.0244664 (PMC7909642; doi:10.1371/journal.pone.0244664)
Supplement: Attachment — Submitted filename: Response to Reviewers.docx [file pone.0244664.s006.docx]

December 8, 2020

Re: Resubmission of manuscript, “Exploring determinants of hydrocele surgery coverage related to Lymphatic Filariasis in Nepal: An implementation research study”, PONE-D-20-21081R1

Yaobi Zhang, M.D., Ph.D.

Academic Editor

PLOS ONE

Dear Dr. Zhang,

Thank you once again for inviting us to submit a revised draft of our manuscript titled, “Exploring determinants of hydrocele surgery coverage related to Lymphatic Filariasis in Nepal: An implementation research study” to PLOS ONE. We really appreciate the time and effort you and each of the reviewers have dedicated by providing insightful feedback to further improve our paper. We have incorporated changes that reflect the suggestions you have graciously provided. We hope that the responses we have provided, and the changes made satisfactorily address all the concerns you and the reviewers have noted.

To facilitate your review of our revisions, point-by-point responses to the comments are provided below.

**REVIEWERS’ COMMENTS:**

Reviewer #2: The authors have done an adequate job of addressing the majority of issues highlighted in the previous version, including all the major concerns raised. A few minor issues remain unaddressed, as well as several novel issues introduced by the revisions.

Minor comments (line numbers refer to the clean version of the revised manuscript):

1. Abstract, line 36: it would be useful to state the type of persons interviewed (e.g. national and district LF focal persons), as “stakeholders” is not defined in the abstract and can refer to a wide variety of individuals both internal and external to the program.

- **Response:** Thank you very much for your insights. We agree with your assessment and have made changes accordingly.

2. Introduction, line 107. The WHO process for LF elimination as a public health problem is “validation”, not “certification”.

- **Response:** Thank you for your assessment. We have made the correction.

3. Introduction, line 107-109: Given that it is now late 2020, the sentence beginning, “Nepal is also gearing-up to achieve LF elimination by 2020; ….” does not seem realistic. Stating MDA has reached 100% coverage is also not relevant toward this goal. What is the percentage districts that have stopped MDA? Completed TAS-3?

- **Response:** Thank you for your valid assessment. We agree with you and have thus revised the section which reflects recent data.

4. Methods (lines 178 and 182): please correct the format for numbers given in “X,XX,XXX” format.

- **Response:** Thank you for your assessment. We have made the corrections accordingly.

5. Methods (line 213-214): “Written and informed consents”. Do the authors mean “Written informed consent”? All consent should be informed consent.

- **Response:** Thank you for your valid assessment. We have corrected the sentence.

6. Discussion (line 684): The statement that “Stakeholders and healthcare providers should consider this issue in order to use the already established network of FCHV….” does not comport with the finding that “gender stigmatization further creates barrier among FCHVs and hydrocele patients”. Suggest revising.

- **Response:** We agree with your assessment and have incorporated changes as reflected in page 29-30, line 685-692.

7. Discussion (line 692): suggest adding “perceived”: “Surgery was avoided in some cases because it was associated with perceived complications such as…..”

- **Response:** Thank you for your valid insight. We agree with you and have made the correction accordingly.

8. The incorrect reference was #12 in the original manuscript; #21 in the revised manuscript. The correct citation is Thomas et al., not Jindau et al.

- **Response**: Thank you for this important insight. We agree with you and have thus made corrections.

Thank you once again for giving us the opportunity to strengthen our manuscript with your valuable comments and suggestions. We have worked hard to incorporate your feedback and hope that these revisions persuade you to accept our submission.

Sincerely,

Choden Lama Yonzon

Corresponding Author

Universitas Gadjah Mada

Yogyakarta, Indonesia

[yonzon.chhoden.cy@gmail.com](mailto:yonzon.chhoden.cy@gmail.com)
